# Paediatric Palliative Care in Resource-Poor Countries

**DOI:** 10.3390/children5020027

**Published:** 2018-02-19

**Authors:** Julia Downing, Sue Boucher, Alex Daniels, Busi Nkosi

**Affiliations:** International Children’s Palliative Care Network, Assagay 3624, South Africa; sue.boucher@icpcn.org (S.B.); education@icpcn.org (A.D.); busi.nkosi@icpcn.org (B.N.)

**Keywords:** palliative care, paediatric/pediatric/children, low-resource settings, advocacy, education, access to medicines

## Abstract

There is a great need for paediatric palliative care (PPC) services globally, but access to services is lacking in many parts of the world, particularly in resource-poor settings. Globally it is estimated that 21.6 million children need access to palliative care, with 8.2 needing specialist services. PC has been identified as important within the global health agenda e.g., within universal health coverage, and a recent Lancet commission report recognised the need for PPC. However, a variety of challenges have been identified to PPC development globally such as: access to treatment, access to medications such as oral morphine, opiophobia, a lack of trained health and social care professionals, a lack of PPC policies and a lack of awareness about PPC. These challenges can be overcome utilising a variety of strategies including advocacy and public awareness, education, access to medications, implementation and research. Examples will be discussed impacting on the provision of PPC in resource-poor settings. High-quality PPC service provision can be provided with resource-poor settings, and there is an urgent need to scale up affordable, accessible, and quality PPC services globally to ensure that all children needing palliative care can access it.

## 1. Introduction

There is a need for paediatric palliative care (PPC) services around the world, yet access to services remains intermittent and at times non-existent, particularly in many Lower- and Middle-Income Countries (LMICs). However, there are various good quality services in LMICs that have successfully overcome some of the challenges to provision. This paper will explore the need for PPC globally, and particularly in LMICs, along with challenges to service provision and how these challenges can be overcome through advocacy and public awareness, education, access to medications, implementation and research.

## 2. The Need for Paediatric Palliative Care

At least 26% of the global population is under 15 years of age, rising to up to 40% in low-resourced settings such as sub-Saharan Africa [1]. Yet, the need for PPC has not been recognised in many LMICs, where development has lagged behind that of palliative care for adults. The World Health Organization (WHO) [2] states that *“palliative care for children represents a special, albeit closely related field to adult palliative care”*.

Palliative care for children is the active, total care of the child’s body, mind and spirit, and also involves giving support to the family.

- It begins when illness is diagnosed and continues regardless of whether or not a child receives treatment directed at the disease.
- Health providers must evaluate and alleviate a child’s physical, psychological and social distress.
- Effective palliative care requires a broad multidisciplinary approach that includes the family and makes use of available community resources; it can be successfully implemented even if resources are limited.
- It can be provided in tertiary care facilities, in community health centres and even in children’s homes.

Other definitions exist, such as that from Together for Short Lives [3], which states that “palliative care for children and young people with life-limiting conditions is an active and total approach to care, from the point of diagnosis or recognition, embracing physical, emotional, social and spiritual elements through to death and beyond. It focuses on enhancement of quality of life for the child/young person and support for the family and includes the management of distressing symptoms, provision of short breaks and care through death and bereavement.” Whilst the Lancet Commission on Pain and Palliative Care built on the WHO definition and adopted the term ‘Serious Health Related Suffering’ (SHS) [4], the WHO definition is that which is generally used within most resource-poor settings, and in many key documents, such as the 2014 World Health Assembly (WHA) Resolution on Palliative Care [5] and within the WHO definition of Universal Health Coverage (UHC) [6]. 

The Global Atlas of Palliative Care at the End of Life [7] estimates that globally, more than seven million children need palliative care at the end of life. Out of these seven million, nearly half of them (49%) are in Africa with 97% of all children needing palliative care at the end of life living in low-resourced settings, i.e., LMICs. Importantly, the Global Atlas notes that of the seven million needing palliative care at the end of life, 67.7% will die from perinatal conditions, 9.8% from congenital anomalies, 6.5% from Human Immunodeficiency Virus (HIV)/Acquired Immune Deficiency Syndrome (AIDS) and only 3.1% from malignant neoplasms. Thus, despite external perceptions, within PPC the majority of children needing palliative care will not have cancer, but other life-limiting and life-threatening conditions. Work has been carried out, mainly in well-resourced settings to look at the types of children requiring palliative care, with Hain et al. [8] identifying more than 376 conditions from death certificates in the UK, with 32% of deaths being from the ten most common diagnosis. In addition, Together for Short Lives [9] has developed a classification system of four categories of life-limiting and life-threatening conditions in childhood that would benefit from palliative care service provision, based largely on disease trajectories, to facilitate service provision provided around need (Table 1). Wood et al. [10] have further built on these categories by the inclusion of two further categories—that of neonates with limited life expectancy and family members who have unexpectedly lost a child. However, there is ongoing discussion as to whether extra categories are needed, for example, whilst bereavement care is important, bereavement in itself is not a life-limiting category. Whilst this debate may continue, the important thing is not the category, but whether all children who need palliative care, and their families, can access it regardless of their condition.

**Figure 1:**
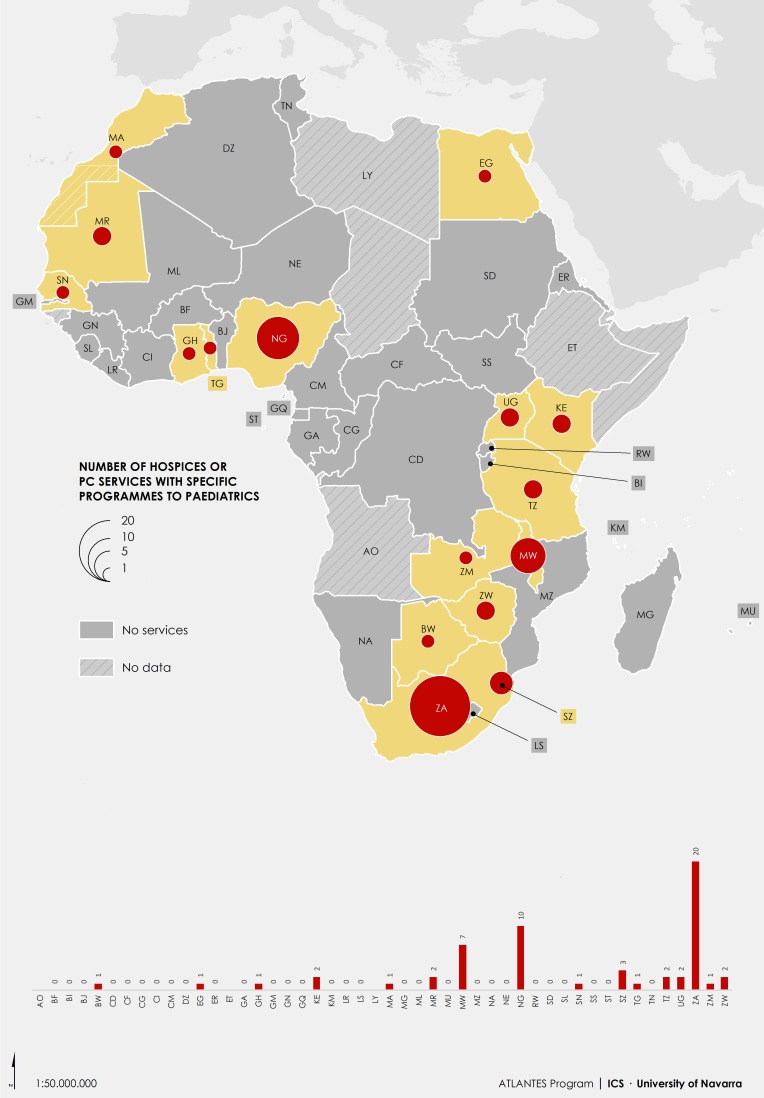
Number of Paediatric Palliative Care (PPC) programmes across Africa ([15] p. 37).

More recently, a study published in 2017 [11] looked beyond the end-of-life and mortality figures and estimated the global need for PPC to be 21.6 million, with 8.2 million children needing access to specialist palliative care service provision. Need ranged between countries from 21 per 10,000 to >100 per 10,000 [11,12,13] with the greatest need being seen in low-resourced settings and those with high rates of HIV. The Lancet Commission report [4] addressed the issue of Serious Health-related Suffering (SHS) noting that one third of children aged 15 years or younger who died in 2015 (almost 2.5 million) experienced SHS and >98% of these lived in LMICs. However, a review of PPC services in 2011 [14] found that despite need, 65.6% of countries had no known PPC activity, with only 5.7% having provision reaching mainstream providers and these were well-resourced countries. Likewise, the APCA Atlas of Palliative Care in Africa [15], and the Atlas of Palliative Care in the Eastern Mediterranean Region [16] both demonstrated this ongoing lack of PPC service provision (Figure 1). 

Studies have shown that strengthening palliative care is a key component of strengthening healthcare systems more generally, as the impact of changing attitudes, skills and knowledge extends beyond that of palliative care itself [17]. The Sustainable Development Goals (SDGs) set out 17 goals and 168 targets to end extreme poverty, fight inequalities and injustice, and protect our planet by 2030 [18]. SDG 3 is about ‘Good health and well-being’ with palliative care fitting well under this goal, although not specifically identified as a target. However, the recent emphasis on UHC, arguably one of the most pressing issues in healthcare today [19], has brought the spotlight on palliative care. Palliative care is being seen as a component of the essential and needed spectrum of health services as defined within UHC by the WHO [6]. UHC is about providing everyone with adequate health services, which must include palliative care services to seriously ill children, who are arguably some of the most vulnerable people within a population. This was emphasised in the Lancet Commission report ‘Alleviating the access abyss in palliative care and pain relief—an imperative of UHC.’ [4]. Whilst recognising that children who need palliative care face tremendous barriers to accessing it, and removing these barriers must be a priority, and providing some child related recommendations, they call on a future Lancet Commission to focus on the issue of palliative care for children, acknowledging that the area could not be fully covered within the scope of the existing commission. This would open ongoing opportunities for prioritising the global health agenda on PPC. 

## 3. Challenges to PPC Development in LMICs

There are a variety of challenges to developing PPC services, the majority of which impact disproportionately on LMICs [4,20,21]. Whilst exact statistics may not be known due to limitations in data collection, the burden of life-limiting and life-threatening conditions in LMICs is high. For example, in 2016 in sub-Saharan Africa there were 160,000 children <15 years of age infected with HIV [22]. Likewise, the rate of non-communicable diseases, both in adults and children, is set to increase exponentially in LMICs by 2030 [23]. The incidence of childhood cancers globally is also increasing, with 215,000 cases reported in children <14 years in 2016 with many more remaining unreported due to a lack of childhood cancer registries in many countries [24]. Other conditions requiring PPC, such as Multi-drug-resistant tuberculosis (MDR-TB) [25], malaria and perinatal and congenital conditions, are also on the increase; therefore, the need for PPC in LMICs is set to increase, whilst provision remains poor. Alongside this, resources remain limited, both in terms of access to treatments, medications, finances and trained personnel, to name but a few. 

Access to treatment can be a challenge. For example, in Eastern and Southern Africa only 63% of children <15 years living with HIV are accessing antiretroviral therapy [22]. Chemotherapy and other medications for the treatment and management of cancer are limited and at times not available. Across sub-Saharan Africa there are a significant number of countries with no radiotherapy machines, so children needing radiotherapy treatment have to travel outside of their country to access this treatment [26]. Access to analgesics, such as oral morphine, also remains a challenge as highlighted in the Lancet Commission report [4]. There are many children globally who are suffering needlessly due to pain and distressing symptoms because the essential medicines for palliative care are not available [27,28]. For children, it is also important that paediatric formulations are available, along with immediate-release liquid oral morphine [20]. It is important to note that despite current poor access, the cost to cover morphine-equivalent pain treatment for all children <15 years with SHS in low income countries would only be $1 million per year [4]. However, even when oral morphine is available, access can be hindered by ‘opiophobia’. Opiophobia, the fear individuals (both health and non-health care professionals) have about prescribing/using opioids, exists in many parts of the world, with beliefs ranging from there being a maximum dose of morphine that can be given safely, the fear of addiction, of tolerance, of issues of respiratory depression, and of the possibility of misuse, to name but a few [29], with these fears frequently being heightened in PPC, alongside many commonly held myths about pain in children, such as babies not experiencing pain [20].

More barriers to the development of PPC include a lack of related policies and guidelines. Whilst countries will have policies for HIV and Non Communicable Diseases (NCDs), these may or may not include the provision of palliative care, and the majority of LMICs do not have a specific palliative care policy. While there has been some progress in this area, with countries such as Botswana, South Africa, Rwanda, Malawi, Uganda and Kenya leading the way, without specific policies, palliative care for a population, including children, may not appear in a government’s work plan or budget. It is therefore essential that PPC is integrated into a broad range of national policies as well as in specific national palliative care policies. Clinical guidelines also need to be available for use in LMICs, utilising appropriate and available medications, without compromising the quality of the care provided.

The lack of trained health and social care workers in PPC is another issue that needs to be addressed. Whilst the WHA resolution [5], along with the European Association of Palliative Care (EAPC) core competencies for PPC [30,31], recognise the need for education in PPC at three levels, such training is not available in the majority of LMICs, with the lack of education and trained professionals being a major barrier to care provision [32,33]. Alongside this, there is a general lack of awareness of what palliative care entails, the impact it can have, and insufficient acknowledgement of the need for PPC, both at the community and the policy levels. Finally, lack of financial resources, in terms of education, research and clinical care, remains a significant barrier to the growth of PPC provision [20]. Funding for PPC in LMICs is limited and donors willing to fund this type of service have reduced over the past few years, making the situation even more critical [4]. 

## 4. Overcoming Barriers and Challenges to PPC Provision

### 4.1. Advocacy and Public Awareness

Advocacy is a key component of any development work for PPC. Advocacy is needed at all levels of the health system: from within local communities, at hospitals and health centres, at governmental level, within law enforcement agencies, with policy makers, and internationally in order to ensure that there is an urgent and universal agenda promoting the ongoing development of PPC service provision. 

Examples of such advocacy work for PPC is seen throughout LMICs where PPC is developing. Advocacy at the community level allows the community, including patients and families, to become aware of what palliative care is and how it can support and help them when it is needed and also gives guidance on how they can become involved. This is an essential element of advocacy work which will assist in overcoming challenges such as a lack of community ownership for PPC programmes, challenging the myths about opioid use and enabling home care support. Community ownership is key and was recognised as one of the core components of a successful model of PPC in sub-Saharan Africa [34]. 

At the national level, work is ongoing with governments, law enforcement agencies and multi-lateral and national organisations. Recognition of PPC at the national level is essential if there is to be ongoing planning and support (both in terms of resources, personnel and finances) for PPC service provision. Likewise, holding governments to account for some of the international resolutions and treaties they have signed, such as the WHA Resolution on PC [5] and Cancer Care [35], is important and can be a way to hold governments to account while offering to support them to fulfil their obligations and having something to report back on at the next WHA.

Regional and international palliative care organisations are committed to advocating for the development of PC and PPC services. Leading the advocacy activities within the regions are the APCA, the EAPC, the Asia Pacific Hospice and Palliative Care Network (APHN) and the Latin American Association for Palliative Care (ALCP), all of which have key roles in bringing together the thoughts of the region, and bringing their concerns and interests to the international level. They are an important forum in supporting national palliative care associations to advocate with governments and encouraging them to support different motions and resolutions at key meetings. The International Children’s Palliative Care Network (ICPCN), the Worldwide Hospice Palliative Care Alliance (WHPCA) and the International Association of Hospice and Palliative Care (IAHPC) collaborate at the international level to bring the global voice of palliative care to the table, with the ICPCN advocating for the voice of the child and PPC. Bringing together the collaborative voice has been successful in supporting side-events at the WHA, the WHA resolution on Palliative care [5] and the WHA resolution on cancer care [35], to name but a few. 

Working with multi-lateral organisations such as the WHO and the United Nations Children’s Fund (UNICEF) is also important, ensuring that the palliative care voice is heard within issues such as UHC [6], the SDGs [18] and efforts to improve access to medications [4]. Work has been undertaken with human rights organisations such as Human Rights Watch [36] to ensure that access to medications is on the global agenda, with access to pain control and palliative care being seen as an essential human right [37,38]. Thus, the development of palliative care should be seen as a public health and human rights priority. Other important documents and advocacy activities include World Hospice and Palliative Care Day, celebrated on the second Saturday in October each year, with emphasis being placed on key global issues such as the lack of access to UHC [19]. Work has also been undertaken on developing a PC essential medicines list [27,28], essential practices for primary palliative care [39] and the recent Lancet Commission report on an essential package of health care interventions [4]. 

### 4.2. Education

Education at all levels of health and social care professional training is essential for the ongoing development of PPC. In line with recent recommendations, such as those within the WHA resolution [5] and the Lancet Commission report [4], education on PPC should be provided across three levels of training: (a) the Palliative Care Approach; (b) General PPC; and (c) Specialist PPC [30,31]. The EAPC developed core competencies for PPC training which were aimed at these three levels and are useful across a range of different settings and countries [30,31].

The first level of training is that of the palliative care approach, which is aimed at educating undergraduate/pre-registration students as well as qualified professionals so that they can integrate PPC in non-specialist settings [30,31]. A variety of competency documents e.g., the EAPC Guide for the Development of Palliative Nurse Education [40], the APCA Core competencies document [41], have been developed to support this level of education. Examples of integrating PC generally, and PPC as appropriate, can be seen from a variety of different LMICs such as in Uganda [42] and Serbia [43]. If PPC can be integrated at this level, then all future health and social care professionals will qualify with an element of PPC knowledge. 

General PPC training is for those who are more frequently involved in PPC but do not provide it as the main part of their work [30,31]. A wide variety of curricula and programmes have been developed for this level of training, which is essential to overcome some of the myths and barriers to PPC provision. Examples of such training include the ICPCN elearning programmes [44], the Education in Palliative and End-of-Life Care (EPEC) Paediatric curriculum [45], the PC toolkit training materials [46], the ecancer elearning programmes focused on sub-Saharan Africa and India [47], along with a wide range of locally developed courses. Ensuring that individuals are provided with this level of training will help in providing the continuum of care for individuals, and will support those providing specialist PPC services. For example, the link nurse programme developed at Mulago Hospital in Uganda, and now expanded to other hospitals, ensures that nurses on the wards throughout the hospital can provide a generalist level of PC provision, including PPC, and thus support the specialist team, with 80% of patients being seen by the link nurses and 20% by the specialist team [48].

Specialist palliative care education is aimed at those for whom the provision of PPC is their core activity. Programmes at this level may be focused specifically on different cadres e.g., doctors or nurses, or they may be multi-professional. There are limited numbers of specialist training programmes available in LMICs for PPC, although examples include paediatric PC training for doctors in Argentina, the Masters/Postgraduate Diploma in PC (Paediatric) provided through the University of Cape Town and the Diploma in PPC from Mildmay Uganda [42]. There is a great need to increase specialist level training—ensuring that it is both accessible and affordable, as well as culturally appropriate, thus increasing the number of PPC specialists in LMICs. An essential ingredient of any training, regardless of at which level, is that of clinical modelling so that professionals can see PPC in practice. However, there remain limited environments where this is possible.

Much of the provision of PPC in LMICs is done in the community, so it is also important to train community health workers on PPC. An example of such training has been in South Africa where training for community health workers on PPC has enabled the expansion of PPC provision. Likewise, parents and family members need training in order to support them to look after their child.

### 4.3. Access to Medications

Access to essential palliative care medicines, such as oral morphine, is limited in many countries and indeed the recent Lancet Commission report focused on this and the disparities seen throughout the world in terms of both availability and price variations [4]. Both the WHO [28] and the IAHPC [27] have accepted essential medicines lists of palliative care, and the Lancet report also recommends an essential package that includes medications; however, this remains an issue in many places. In addition, some countries may have secured access to medications for adults but paediatric formulations are not yet available. There is ongoing work at the international, regional and national levels to address the issue of access to medications, ensuring that they will be both available, but also accessible to all children who need them, regardless of where they live. The ICPCN, IAHPC and WHPCA continue to try and address this issue, working closely with some of the key stakeholders, including the International Narcotics Control Board, the United Nations (UN) Councils, the WHO, etc. This situation has been impacted by the current situation in the United States of America (USA) regarding the misuse of medications, which was highlighted in the Lancet Commission report. There are examples in a wide range of LMICs of how access to essential medicines for PPC has been improved e.g., in Kenya, in South Africa and in Zambia.

### 4.4. Implementation of PPC

A variety of models of PPC delivery exist within LMICs, covering the continuum of care. Home-based care is important, enabling families to care for their children at home, in their own environment, with the support of community health workers and volunteers. Rachael House in Indonesia is a good example of how a team can work within the existing health care structure to support children with palliative care needs, and their families, at home [20]. However, home-based care is just one model of providing PPC and it is important that care is provided across different settings, in accordance to need, and the WHO definition of PPC. Other models include hospital PPC teams, outpatient care, day care and inpatient care [49,50]. Examples of each of these exist with the LMIC setting, for example Mildmay Uganda have an inpatient as well as day care and outpatient services [51], Umodzi, at Queen Elizabeth Hospital in Blantrye, in Malawi is an example of a specialist PPC hospital team [20,52]. 

**Figure 2:**
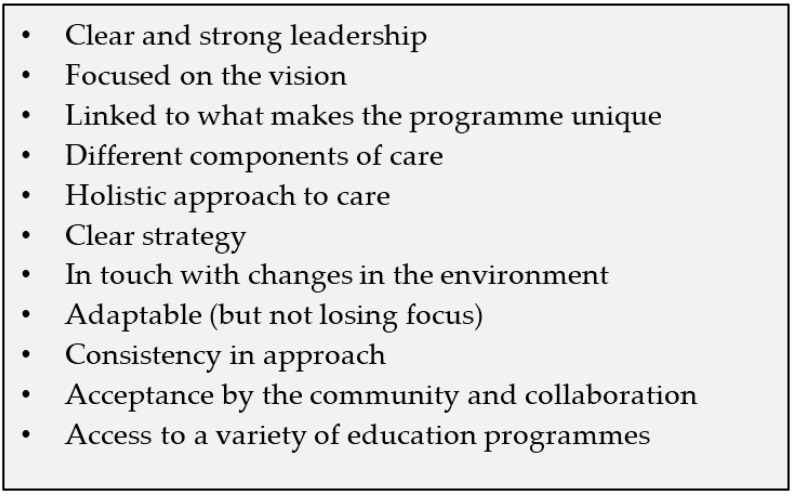
Key elements of an effective PPC programme [34].

Significant challenges exist to the provision of PPC in remote rural areas, and work has been done looking at the provision of such services in different settings in order to draw on the lessons learnt [53]. Centres of Excellence or Beacon Centres are being developed in different settings in order to not only provide PPC services, but also to provide an environment for clinical placements and learning [54]. Some work was undertaken across sub-Saharan Africa to identify the key areas for an effective PPC programme (Figure 2) and to try and understand the challenges faced by different PPC service providers. Challenges to service provision included a lack of financial resources; the high disease burden; lack of collaboration, PPC not being a priority; poor integration into the formal health services; and staffing issues. It was found that whilst sharing common core elements, each service had unique aspects that were important and ensured they were appropriate and embedded into their local community and context. Successful PPC services could also be integrated into or run parallel with existing structures, depending on the system in which they are operating [34].

### 4.5. Research

Whilst originally not seen as a priority, the importance of research has been recognised in developing the evidence base, and seen as a key pillar in palliative care development [55] alongside policy, drug availability, education and implementation [56]. Whilst resources for service delivery may be limited in many LMICs, it is essential that we undertake research to ensure that we are using those resources wisely, and adapting them as required. However, there has been a lack of research in palliative care generally [57], and more specifically in PPC. In 2010, just five peer-reviewed papers were found on PPC in sub-Saharan Africa, and a report on the status of PPC across the region highlighted that the evidence base for PPC had not progressed, there were no measurement tools, few models were discussed and that there was an urgent need for research within the field [58]. 

**Figure 3:**
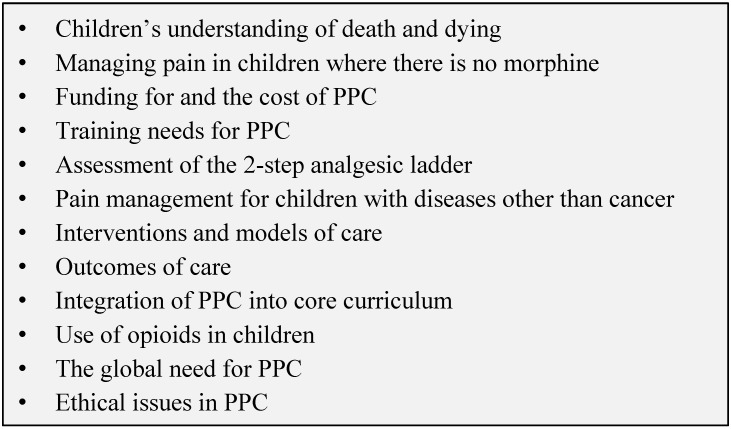
Global Priorities for PPC Research [61].

This lack of research has been identified on many occasions over the years [4,58,59,60,61,62]. The WHO guidelines on the pharmacological management of persisting pain in children with medical illnesses [63] highlight how this lack of evidence has impacted on the development of evidence-based guidelines, and therefore the management of pain within PPC, and proceed to make a call for the expansion of the evidence base within the field [62]. Global priorities for research on PPC have been identified which encompass holistic care, education, interventions and models, legislation and ethics and policies and procedures (Figure 3). Thus, the generation of evidence needs to be recognised as an integral part of PPC service provision to ensure that we can deliver evidence-based, cost-effective and equitable PPC, not just in LMICs, but globally [61].

## 5. Conclusions

Many examples of high-quality PPC service provision within LMICs exist; however, these remain few and far between, with an urgent need to scale up affordable, accessible and quality PC services for children globally. Whilst challenges exist inhibiting the ongoing development of PPC, these have been identified, and work is ongoing in different contexts and at different levels, to try and overcome these barriers. Strategies should be put in place to continue to scale up PPC service provision in LMICs, so that access is assured for all children who need palliative care, and their families, regardless of where they live.

## Figures and Tables

**Table 1:** Categories of life-limiting and life-threatening conditions [9].

Category 1	Those children with life-threatening conditions for which curative treatment may be feasible but can fail. e.g., cancer, irreversible organ failure
Category 2	Those children with conditions in which there may be long phases of intensive treatment aimed at prolonging life, but premature death is still possible e.g., cystic fibrosis, Duchenne muscular dystrophy
Category 3	Those children with progressive conditions without curative treatment. e.g., batten disease, mucopolysaccharidoses
Category 4	Those children with conditions with severe neurological disability, which may deteriorate unpredictably, but are not considered progressive.
